# Antiangiogenic Activity of 2-Deoxy-D-Glucose

**DOI:** 10.1371/journal.pone.0013699

**Published:** 2010-10-27

**Authors:** Jaime R. Merchan, Krisztina Kovács, Jaclyn W. Railsback, Metin Kurtoglu, Yuqi Jing, Yolanda Piña, Ningguo Gao, Timothy G. Murray, Mark A. Lehrman, Theodore J. Lampidis

**Affiliations:** 1 Department of Medicine, University of Miami Miller School of Medicine and Sylvester Comprehensive Cancer Center, Miami, Florida, United States of America; 2 Department of Cell Biology, University of Miami Miller School of Medicine and Sylvester Comprehensive Cancer Center, Miami, Florida, United States of America; 3 Department of Ophthalmology, Bascom Palmer Eye Institute/University of Miami Miller School of Medicine, Miami, Florida, United States of America; 4 Department of Pharmacology, University of Texas Southwestern Medical Center at Dallas, Dallas, Texas, United States of America; Universidade de São Paulo, Brazil

## Abstract

**Background:**

During tumor angiogenesis, endothelial cells (ECs) are engaged in a number of energy consuming biological processes, such as proliferation, migration, and capillary formation. Since glucose uptake and metabolism are increased to meet this energy need, the effects of the glycolytic inhibitor 2-deoxy-D-glucose (2-DG) on *in vitro* and *in vivo* angiogenesis were investigated.

**Methodology/Principal Findings:**

In cell culture, 2-DG inhibited EC growth, induced cytotoxicity, blocked migration, and inhibited actively forming but not established endothelial capillaries. Surprisingly, 2-DG was a better inhibitor of these EC properties than two more efficacious glycolytic inhibitors, 2-fluorodeoxy-D-glucose and oxamate. As an alternative to a glycolytic inhibitory mechanism, we considered 2-DG's ability to interfere with endothelial N-linked glycosylation. 2-DG's effects were reversed by mannose, an N-linked glycosylation precursor, and at relevant concentrations 2-DG also inhibited synthesis of the lipid linked oligosaccharide (LLO) N-glycosylation donor in a mannose-reversible manner. Inhibition of LLO synthesis activated the unfolded protein response (UPR), which resulted in induction of GADD153/CHOP and EC apoptosis (TUNEL assay). Thus, 2-DG's effects on ECs appeared primarily due to inhibition of LLOs synthesis, not glycolysis. 2-DG was then evaluated in two mouse models, inhibiting angiogenesis in both the matrigel plug assay and the LH_BETA_T_AG_ transgenic retinoblastoma model.

**Conclusions/Significance:**

In conclusion, 2-DG inhibits endothelial cell angiogenesis *in vitro* and *in vivo*, at concentrations below those affecting tumor cells directly, most likely by interfering with N-linked glycosylation rather than glycolysis. Our data underscore the importance of glucose metabolism on neovascularization, and demonstrate a novel approach for anti-angiogenic strategies.

## Introduction

Angiogenesis – the process of new blood vessel growth - is critical for several physiological and pathological processes, such as cancer, autoimmune diseases, age related macular degeneration and atherosclerosis, among others [1], [2]. The process of tumor angiogenesis involves activation of ECs by angiogenic growth factors, such as vascular endothelial growth factor (VEGF), or basic fibroblast growth factor (bFGF). These factors induce EC proliferation, migration, and organization into new capillaries, which require energy in the form of ATP. ATP generation in ECs has been shown to derive mainly from glucose uptake and utilization [3], [4], [5].

A common property of invasive and metastatic tumors is upregulation of glycolysis, leading to enhanced glucose consumption [6], [7]. Upregulation of glycolysis in tumors is mediated by activation of oncogenes, loss of tumor suppressors, or by adaptive responses to hypoxia in the tumor microenvironment [8]. The avid uptake of glucose by tumors is the foundation for functional tumor imaging by fluoro-deoxyglucose positron emission tomography (PET) [7], [9].

Preclinical and clinical studies have suggested that activated or pathologic endothelium is associated with enhanced glucose uptake. VEGF and hypoxia induce EC expression of glucose transporters and uptake [9], [10], [11], [12]. Clinical reports show that in conditions associated with vascular injury and inflammation, diseased vessels have significantly increased uptake of 2-FDG [13], [14]. Moreover, in breast cancer, a positive correlation exists between microvessel density and 2-FDG uptake in tumors [15]. Since the endothelium represents an important portion of the tumor stroma, it has been suggested that tumor vasculature may contribute to 2-FDG uptake in tumors [16]. Endothelial glucose metabolism therefore may represent a novel target for angiogenesis inhibition.

2-deoxy-D-Glucose (2-DG) is a sugar analog that interferes with glycolysis and glycosylation [17], [18], and has been shown to induce *in vitro* and *in vivo* antitumor effects in combination with chemotherapy [19], [20], [21], [22]. Furthermore, safety and feasibility of oral 2-DG administration has been tested in early clinical trials in cancer patients, as a single agent [23], in combination with chemotherapy [24], or with radiation therapy [25]. To our knowledge, in this report, we present for the first time data that 2-DG significantly inhibits angiogenesis *in vitro* and *in vivo*, but surprisingly appears to do so by a mechanism not primarily dependent upon glycolysis inhibition.

## Materials and Methods

### Cell lines and reagents

2-DG, 2-FDG, oxamate, mannose, and FITC-dextran were purchased from Sigma-Aldrich (St. Louis MO). Matrigel was obtained from BD Biosciences (Bedford, MA) and used *in vitro* at a 7 mg/mL and *in vivo* at a ∼20 mg/mL concentration. The growth factors bFGF and VEGF were purchased from R&D Systems (Minneapolis, MN). Human umbilical vein endothelial cells (HUVECs), human microvascular endothelial cells from lung (HMVEC-L), EGM-2 and EGM2-MV medium were purchased from Lonza (Walkersville, MD). EGM-2 and EGM2-MV contain serum and the following growth factors: hEGF, VEGF, hFGF-B, R3-IGF-1. All other cancer cell lines were purchased from the American Type Culture Collection (ATCC). The cells were cultured according to the supplier's instructions. For western blotting, anti-KDEL for GRP78 and GRP94 was purchased from Stressgen, (Ann Arbor, MI), polyclonal anti-CHOP/GADD153 was purchased from Santa Cruz Biotechnology (Santa Cruz, CA), and polyclonal cleaved Caspase-3 antibody was purchased from Cell Signaling (Danvers, MA). For immunohistochemistry CD31 monoclonal antibody was purchased at BD Bioscience (Bedford, MA).

### Cell Viability and Cytotoxicity assays

A total of 5×10^4^ cells in 1 ml of appropriate medium (specific for each cell line, see above) were seeded into each of a 12 well plate and treated at different concentrations of drugs. Cell culture medium contained 1 mg/ml of glucose. Cells were incubated at 37°C in 5% CO_2_ for different time points (24, 48, or 72 hours). At the end of this period, cells were harvested and viability and cytotoxicity were analyzed by Vi-Cell (Beckman Coulter, Fullerton, CA) cell viability analyzer as previously described [21]. For endothelial cell viability assays, cells were incubated in 1% FBS and stimulated with bFGF (10 ng/ml), unless indicated otherwise.

### Matrigel Tube Formation Assay

The matrigel tube formation was performed as previously described [26], [27]. Each well of a pre-chilled 48-well cell culture plate was coated with 100 µL of unpolymerized Matrigel (7 mg/mL) and incubated at 37°C in 5% CO_2_ for 30–45 minutes. HUVECs were harvested with trypsin, and 4×10^4^ cells were resuspended in 300 µL complete endothelial cell growth medium and treated with the various agents (2-DG, 2-FDG, oxamate, and mannose) at different concentration before plating onto the Matrigel-coated plates. In a separate experiment to assess whether or not 2-DG affected already formed capillaries, HUVECs were plated in complete endothelial cell growth medium and treated with 2-DG after tubes formed (approximately 16–18 hours later). After approximately 24 hours of incubation at 37°C in 5% CO_2_, endothelial cell tube formation was assessed with an inverted photomicroscope (Nikon, Melville, NY). Microphotographs of the center of each were taken at 40X magnification with the aid of imaging-capture software (NIS-Elements from Nikon, Melville, NY). Tube formation in the microphotographs was quantitatively analyzed (total tube length); controls consisted of HUVECs in complete endothelial cell medium. The experiment was done in triplicate and the data presented represent the average of triplicate experiments.

### Migration Scratch Assay

Endothelial migration was assessed by the scratch assay, as previously reported [28]. Briefly, a total of 1×10^5^ HUVECs were seeded -in full endothelial growth medium- in 6-well plates and allowed to form a monolayer overnight in a 37°C in 5% CO_2_ incubator. Using a p200 pipette tip, scratches were made in triplicate in each well of the confluent monolayer. The medium was changed and the wells were treated with different concentrations of 2-DG. The control well was untreated. Microphotographs of the scratches were taken at 0 hours right after scratching the monolayer. Cells were allowed to migrate for 24 hours and a second microphotograph was taken of each scratch to determine the percent of migration of treated cells relative to the 0 hour pictures by quantification with NIS-Elements software (Nikon, Melville, NY). Migration was quantitated by measuring the width of the cell free zone at the time of the scratch (0 hours) and 24 hours after the scratch. Changes in migration on treated cells were expressed as percentage of the (untreated) controls. Values represent the mean (+/− SD) of triplicate scratches.

### Fluorophore-assisted carbohydrate electrophoresis (FACE)

FACE analysis was performed as previously described (22). Briefly, cells were cultured until 90% confluent, received different treatments, and incubated for 24 hours. After this period, cells were harvested in methanol, and dried under vacuum. Lipid linked oligosaccharides (LLOs) were recovered in chloroform/methanol/water (10∶10∶3). The glycans were released from pyrophosphate-linkage to dolichol by mild acid hydrolysis, modified with the fluorophore 7-amino-1,3-naphthalenedisulfonic acid (ANDS, Invitrogen) by reductive amination, resolved on high-percentage polyacrylamide gels, and detected under ultraviolet light with a Biorad Fluor-S charged-coupled device imager as previously described (22). Individual ANDS-labeled glycans were quantified, or alternatively scans (tracings) of all glycans in gel lanes were generated electronically, with the Quantity-One software supplied with the imager.

### Western Blot analysis

Cells were plated with and without drug treatment for the indicated times. At the end of the treatment periods, cells were collected and processed as previously described [29]. Gels were transferred to nitrocellulose membranes (Amersham, Piscataway, NJ) and probed with anti-KDEL (Stressgen, Ann Arbor, MI) for GRP78 and GRP94; polyclonal anti-CHOP/GADD153 (Santa Cruz Biotechnology, Santa Cruz, CA) and polyclonal anti-cleaved Caspase-3 (Cell Signaling, Danvers, MA). Following probing, membranes were processed as previously described [29].

### Terminal deoxynucleotidyl transferase dUTP nick end labeling (TUNEL) assay

HUVEC cells were plated in chamber slides (VWR) at 80 000 cells per chamber, treated with different reagents as indicated and incubated for 24, 48, and 72 hrs. At each time point, apoptosis was detected with an in situ cell death detection kit (Roche Applied Science, Indianapolis, IN), according to the manufacturer's protocol. Apoptosis was quantitatively analyzed by determination of the percentage of TUNEL positive (identified by FITC) cells over total cells (determined by DAPI), using the high content screening Cellomics ArrayScan VTI (Thermo Fisher Scientific, Pittsburg, PA). Eighteen fields per slide (in triplicate) were analyzed for each condition. Results were expressed as percentage of TUNEL positive cells (over total cells) and normalized to control. Experiments were performed in triplicate and repeated at least twice.

### *In vivo* Angiogenesis (Matrigel plug) Assay

Animal protocols were reviewed and approved by the University of Miami animal care and use committee. The Matrigel plug assay was performed as previously described [27], [30], [31], [32], [33] with modifications. Briefly, 500 uL of unpolymerized Matrigel (∼20 mg/mL), either alone (negative control), mixed with bFGF, VEGF (500 ng/ml each) and glucose (6 mM, positive control), or mixed with bFGF, VEGF and 2-DG (6 mM, treatment group) was injected subcutaneously at the left lower abdominal wall of three groups of BALB/C mice (5-to 6-week old; Jackson Laboratories, Bar Harbor, ME). At day 12, the mice were injected intravenously through the tail vein with 200 uL of FITC-dextran (25 mg/mL, Sigma) and sacrificed 20 minutes later. Quantification of FITC-dextran within the plugs was achieved by incubating the plugs in dispase reagent (Becton Dickinson) overnight, followed by homogenization and centrifugation for 30 seconds at 13,000 r.p.m. Fluorescence readings of the supernatant were taken at 480/520 nm using a Spectra Rainbow plate reader (Tecan US; SLT Lab Instruments, Research Triangle Park, NC) and compared to a standard curve of FITC-dextran. Matrigel plugs from a second group of mice that underwent the above treatments were excised, fixed in 4% paraformaldehyde, embedded in paraffin, sectioned, and stained with H&E and CD 31 (see below). Sections were examined by light microscopy and pictures were taken using a Nikon TE2000-U microscope.

### LH_BETA_T_AG_ mouse model of retinoblastoma

The LH_BETA_T_AG_ transgenic mouse model [34], [35] was used to evaluate the *in vivo* effects of systemic administration of 2-DG on tumor angiogenesis. This transgenic model has been previously characterized, and has histological, ultrastructural, and immunohistochemical characteristics identical to those in human retinoblastoma [34], [35], [36]. 2-DG (500 mg/kg) was administered through intraperitoneal injection into tumor bearing mice, starting at 16 weeks of age, three times a week for 5 weeks (n = 5). Saline was administered using the same method (n = 4). At 21 weeks of age, mice were euthanized and eyes were enucleated at 21 weeks of age and examined for analysis of tumor vasculature.

### Immunohistochemistry

CD31 immunohistochemistry of matrigel plugs was performed as published before [9]. Briefly, sections were deparaffinized and hydrated through a series of graded alcohol steps and washed in phosphate buffered saline. Antigen retrieval was carried out in 0.33 mg/mL protease K for 10 min at 37°C. Endogenous peroxidase activity was quenched with 0.6% hydrogen peroxide in methanol for 15 min. Sections were blocked in 10% rat serum for 1 hr, then incubated with rat anti-mouse anti-CD31 antibody (BD Bioscience Bedford, MA) overnight at 4°C. After washing, an anti-rat biotinylated secondary antibody (ABC Elite Kit) was applied for 30 min, washed, and developed using the Avidin/biotin/HRP method (ABC Elite Kit, Vector Labs, Burlingame, CA) and DAB chromogenic reaction (Vector Labs). Finally, sections were counterstained with Gill 2 Hematoxylin (Richard-Allan Scientific/Thermo Scientific, Waltham, MA) and mounted with Cytoseal XYL (Richard-Allan Scientific/Thermo Scientific).

Determination of tumor vasculature in retinoblastoma samples were performed as previously reported [35]. Tumor samples were frozen in OCT immediately after enucleation and serially sectioned (8 µm). Slides were fixed with methanol for 10 minutes (−20°C) before immunohistochemical analyses. Total vessels were detected with Alexa Fluor 568 conjugated lectin (*Bandeira simplicifolia*, a panendothelial binding agent; 1∶1000; Invitrogen, Carlsbad, CA). Omission of the primary antibody (secondary only) was used as a negative control for nonspecific binding. Cell nuclei were stained for 5 minutes with 4′, 6′ diamidino-2-phenylindole (DAPI, 1∶5000; Invitrogen, Carlsbad, CA). Serial cross sections of eyes containing tumors were examined for the presence of the above described marker with a Leica TCP SP5 laser confocal microscope (Leica Microsystems CMS GmbH, Mannheim, Germany). All images were digitally acquired and recompiled (Photoshop CS; Adobe, San Jose, CA). Sections were viewed at 400X magnification. Pictures of 6 areas of the tumor (2 from the apex, 2 from the base, and 2 from the center) were taken for quantification. Differences in tumor microvessels between the control and the 2-DG treated groups were analyzed by quantification of lectin fluorescence staining (in arbitrary units) from each picture, using the NIS-Elements image analysis software (Nikon).

### Statistical analysis

Data are presented as means and 95% confidence intervals, unless otherwise specified. Differences in means among three or more groups were analyzed by analysis of variance (ANOVA) followed by Tukey-Kramer, Fisher's or Wilcoxon rank sum test. Means between two groups were compared by Student's t test analysis. Differences were considered statistically significant at P<0.05. All statistical tests were two-sided.

## Results

### 2-deoxy-D-glucose (2-DG) inhibits *in vitro* endothelial cell growth and induces cytotoxicity

**Figure 1:**
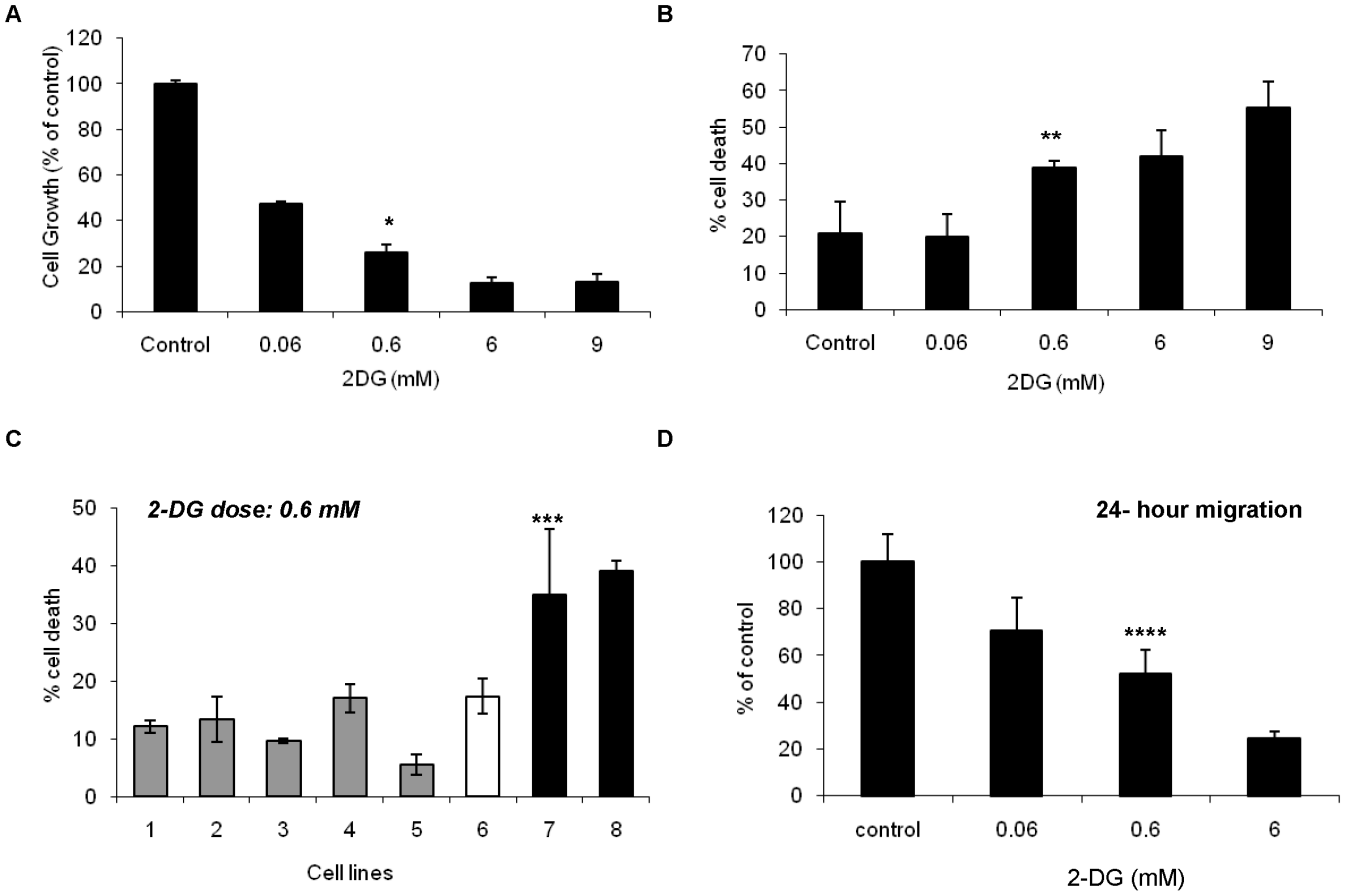
2-DG inhibits endothelial cell growth, migration, and induces endothelial cytotoxicity *in vitro*. HUVECs were stimulated with bFGF (10 ng/ml), treated with different concentrations of 2-DG for 72 hours, and its effects on cell growth (**A**) and cytotoxicity (**B**) were assessed as in materials and methods. **A.** 2-DG significantly inhibited bFGF induced HUVEC cell growth, with 72% inhibition by a concentration of 0.6 mM of 2-DG (* p<0.01, vs. control (0 mM)). Results (percent of control) are presented as the average of triplicate experiments and 95% confidence intervals. **B.** 2-DG induced significant cytotoxic effects on HUVECs in a dose dependent manner. ** p = 0.02; 0.6 mM 2-DG vs. control. **C.** HMVEC-L (lane 7) and HUVECs (lane 8) were significantly more sensitive to the cytotoxic effects of low doses (0.6 mM) of 2-DG than cancer (lanes 1–5) and non-cancer epithelial cells (lane 6). Lane 1: HT-29; 2: CAKI-1; 3: MDA-MB231; 4: 786-0; 5: HT-1080; 6: HREC. *** p<0.001 HUVEC and HMVEC vs. all other cell lines. Results (percent cell death) are presented as the average of triplicate experiments and 95% confidence intervals. **D.** HUVEC migration was assessed by the scratch assay. Significant inhibition of migration at 24 hours was observed, in a dose dependent manner. **** p = 0.009; 0.6 mM 2-DG vs. control. Results (percent of control) are presented as the average and 95% confidence intervals of triplicate experiments. All experiments were repeated at least twice.

Endothelial cell activation by angiogenic growth factors is associated with enhanced glucose transport and utilization [9], [37]. To determine the effects of the glycolytic inhibitor, 2-DG, on the different steps of the angiogenic process, HUVECs were exposed to escalating doses of this sugar analog and *in vitro* growth and cytotoxicity were assessed. 2-DG significantly inhibited bFGF induced HUVEC growth at 72 hr in a dose dependent manner i.e. 52% and 72% inhibition at 0.06 and 0.6 mM, respectively (p = 0.01; Fig. 1A). Cell growth was also impaired at earlier time points (24 and 48 hours), albeit at a lower magnitude as the effects observed at 72 hours (Fig. S1). 2-DG also induced HUVEC cytotoxicity at 72 hours, in a dose dependent manner (Fig. 1B). Significant endothelial cell death (38.7%) was observed starting at doses as low as 0.6 mM of 2-DG (p = 0.02, vs. control). To assess whether EC sensitivity to low concentrations of 2-DG was restricted to HUVECs, cytotoxicity assays were performed on lung derived human microvascular endothelial cells (HMVEC-L) and similar cytotoxic effects were observed (Fig. 1C, lane 7: HMVEC-L and lane 8: HUVEC). Both HUVECs and HMVEC-L cells were significantly more sensitive to the cytotoxic effects of low dose (0.6 mM) 2-DG than a panel of human cancer cells (HT-29, CAKI-1, MDA-MB231, 786-0 and HT-1080), and normal human renal epithelial cells (HREC) (Fig. 1C; p<0.001).

### 2-DG inhibits endothelial capillary formation and endothelial cell migration *in vitro*

After demonstrating that low doses (0.6 mM) of 2-DG inhibit EC growth and induces EC cytotoxicity, the effects of this compound on HUVEC migration were evaluated by the endothelial scratch assay [28]. In this assay, HUVECs were stimulated with full endothelial growth medium which contains 2% serum and angiogenic growth factors (EGM-2, see materials and methods). 2-DG significantly inhibited HUVEC migration at 24 hours in a dose dependent manner, with a 48% inhibition at a 2-DG concentration of 0.6 mM (Fig. 1D; p = 0.007). In contrast to the cytotoxic effects of 2-DG observed at 72 hours, at this earlier time point (24 hours), no significant changes in endothelial cell viability were detected (data not shown).

**Figure 2:**
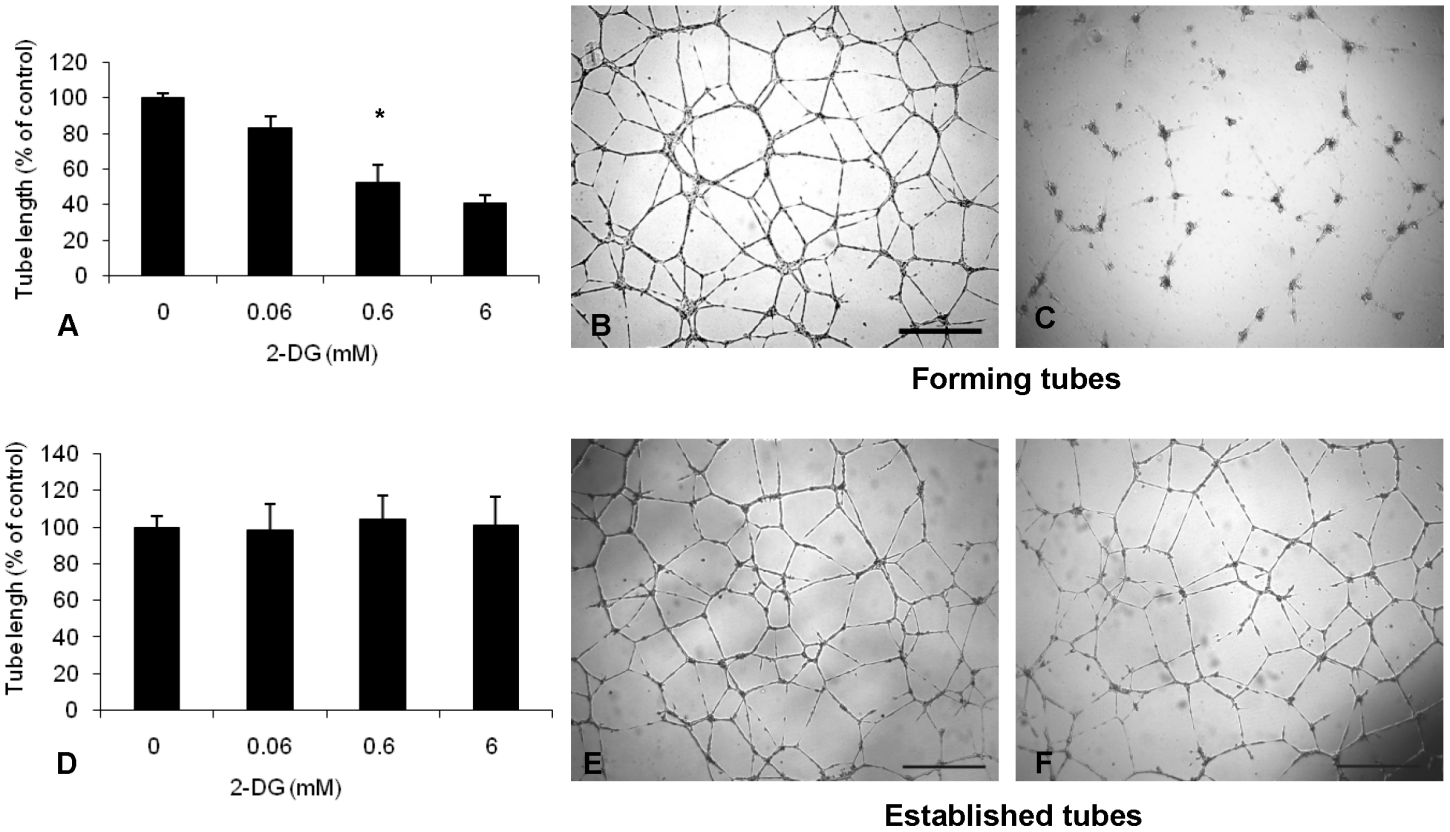
2-DG inhibits HUVEC capillary formation, but does not disrupt already established tubes. HUVECs plated on matrigel were exposed to different concentrations of 2-DG before (upper panel) and after (lower panel) they organized into capillaries. **A.** Significant inhibition of HUVEC tube formation was observed in a dose dependent manner. * p = 0.005, 0.6 mM 2-DG (**C**) compared with control (**B**). In the lower panel, HUVEC capillaries were allowed to form overnight, before they were exposed to 2-DG. Changes in total tube length were assessed 24 hours after 2-DG exposure. **D.** 2-DG did not disrupt already established HUVEC capillaries. **E, F:** Representative pictures of control capillary tubes (**E**) and tubes treated with 0.6 mM 2-DG (**F**). Scale bar = 100 µm.

HUVEC tube formation was also found to be significantly inhibited by 2-DG in a dose dependent manner (Fig. 2A, B, C). Quantification of total tube length showed a 17% (p = 0.008), 48% (p = 0.005), and 59% (p = 0.0009) inhibition of tube formation in HUVECs treated with 2-DG at 0.06 mM, 0.6 mM, and 6 mM, respectively, compared to control. Interestingly, the inhibitory effects of low doses of 2-DG occurred only during active HUVEC capillary formation (Fig. 2A, B, when HUVECs were treated with 2-DG immediately after plating). However, when already established HUVEC capillaries were exposed to 2-DG at doses ranging from 0.06 to 6 mM, no significant capillary disruption was observed (p = NS) (Fig. 2D, E, F).

### 2-DG inhibits *in vitro* angiogenesis more potently than other glycolytic inhibitors and its effects are reversed by mannose treatment

**Figure 3:**
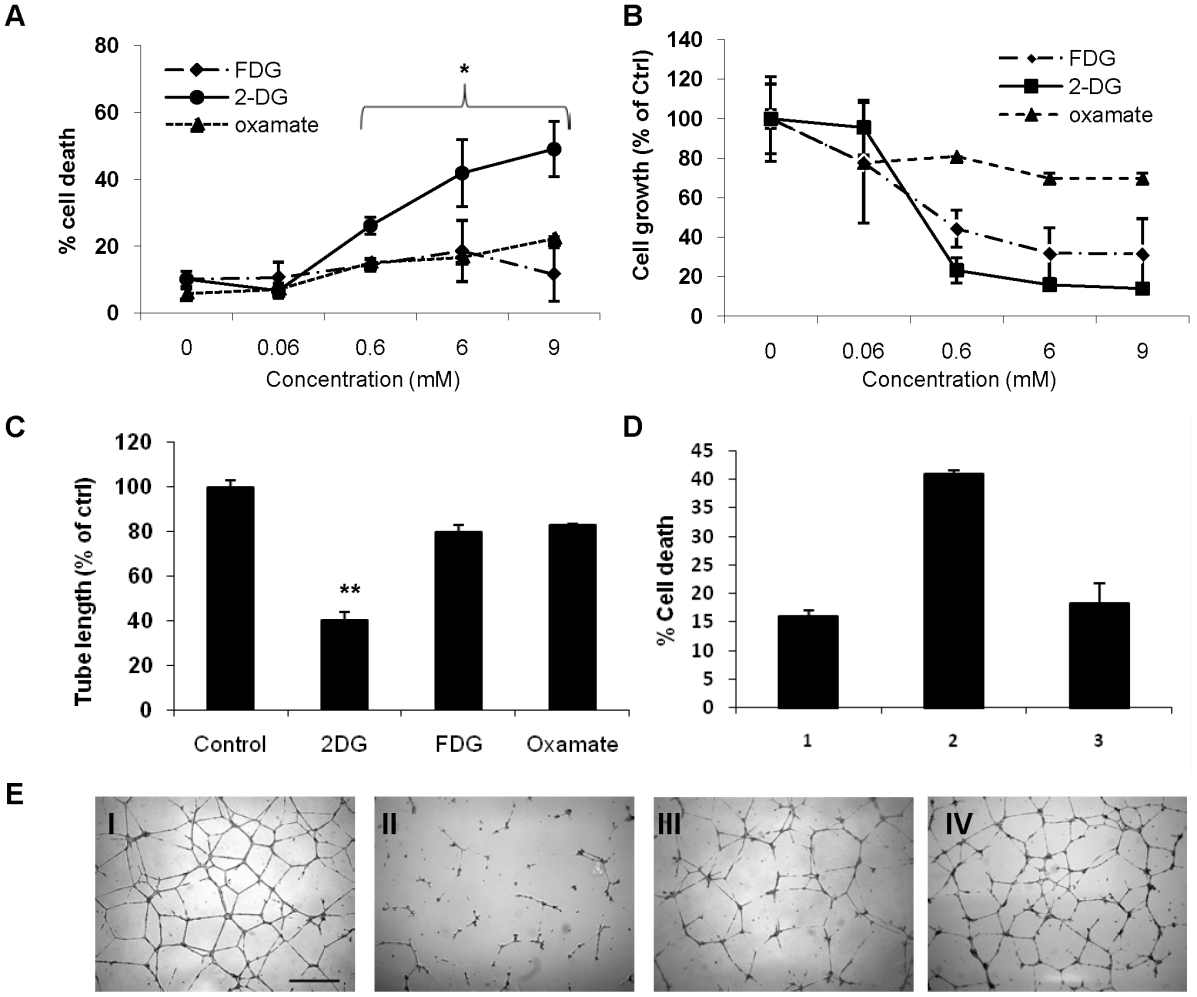
Differential effects of 2-DG and other glycolytic inhibitors on *in vitro* angiogenesis and reversal of 2-DG's antiangiogenic effects by mannose. HUVECs were exposed to 2-DG and the glycolytic inhibitors, 2-FDG and oxamate, and cell growth and cytotoxicity were measured at 72 hours. **A.** 2-DG had significantly more potent cytotoxic effects than 2-FDG and oxamate at equimolar concentrations. * p<0.05, 2-DG vs. 2-FDG and oxamate. **B.** 2-DG and 2-FDG inhibited HUVEC growth more potently than oxamate (p<0.001). The differences between the growth inhibitory effects of 2-DG and 2-FDG were not statistically significant (p>0.5). **C.** HUVECs were exposed to 0.6 mM of 2-DG, 2-FDG and oxamate, and tube formation assay was performed. Quantitative analysis of total tube length was performed as in materials and methods. 2-DG inhibited tube formation more potently than 2-FDG and oxamate. Histogram bars represent the average (and 95% confidence intervals) total tube length (percent of control) of triplicate experiments. ** P<0.0001, 2-DG vs. 2-FDG and oxamate. **D.** Co-treatment of HUVECs with 2-DG and mannose reverted the cytotoxic effects of 2-DG. 1: Control. 2: 2-DG at 0.6 mM. 3: 2-DG (0.6 mM) and mannose (1 mM). **E.** Mannose rescued 2-DG's inhibitory effects on HUVEC tube formation. **I** =  control, **II** = 2-DG (0.6 mM), **III** = 2-DG (0.6 mM) + mannose (1 mM), **IV** =  mannose (1 mM). Scale bar: 100 µm.

The above findings suggest that interfering with endothelial cell glycolysis may explain the effects of 2-DG on in *vitro* angiogenesis. To assess the relative potency of 2-DG as an antiangiogenic agent, the effects of 2-DG were compared to equimolar concentrations of 2-FDG, which has previously been shown to be a better glycolytic inhibitor than 2-DG [18], as well as oxamate (a pure glycolytic inhibitor). 2-FDG and oxamate did not induce significant HUVEC cytotoxicity (p = NS), while 2-DG, as shown above, did induce significant endothelial cell death at concentrations of 0.6, 6 and 9 mM (Fig. 3A; p<0.05). All glycolytic inhibitors interfered with HUVEC growth, albeit the effects of 2-DG and 2-FDG were significantly more potent than oxamate at equimolar concentrations (Fig. 3B; p<0.001, 2-DG and 2-FDG vs. oxamate). Oxamate was associated with mild to moderate (30%) inhibition of EC growth at concentrations of 6 and 9 mM. The effects of 2-DG and 2-FDG on HUVEC growth were not significantly different (p>0.5). When HUVEC tube formation was assayed, the inhibitory effects of low doses (0.6 mM) of 2-DG were significantly more potent than 2-FDG (p<0.0001) and oxamate (p<0.0001) at equimolar concentrations (Fig. 3C). There was no statistically significant difference between 2-FDG and oxamate on their effects on tube formation. These findings strongly suggest that mechanisms other than inhibition of endothelial cell glycolysis may explain the cytotoxic and antiangiogenic effects of 2-DG.

In addition to interfering with glycolysis, 2-DG also interferes with N-linked glycosylation [17], [18]. Due to its similarity in structure to mannose, 2-DG inhibits N-linked glycosylation by competition with mannose metabolism and by fraudulent incorporation into dolichol-pyrophosphate (lipid)–linked oligosaccharides, which are the precursors for N-linked glycosylation [17], [18]. To ascertain whether the antiendothelial effects of low doses of 2-DG might be predominantly due to inhibition of glycosylation, HUVEC cytotoxicity and capillary formation assays were performed in the presence and absence of mannose (1 mM), a sugar previously shown to reverse the effects of 2-DG on N-linked glycosylation in select tumor cells [17], [18]. Mannose potently reversed the 2-DG induced HUVEC cytotoxicity (Fig. 3D) and capillary formation (Fig. 3E). Similar to the results of figure 3, these data are inconsistent with glycolysis as the primary relevant target of 2-DG in endothelial cells, and instead indicate that the above effects are primarily due to interference with endothelial N-linked glycosylation.

### 2-DG interferes with endothelial synthesis of lipid linked oligosaccharides (the precursor of N-linked glycosylation), and induces an ER unfolded protein response and apoptosis

**Figure 4:**
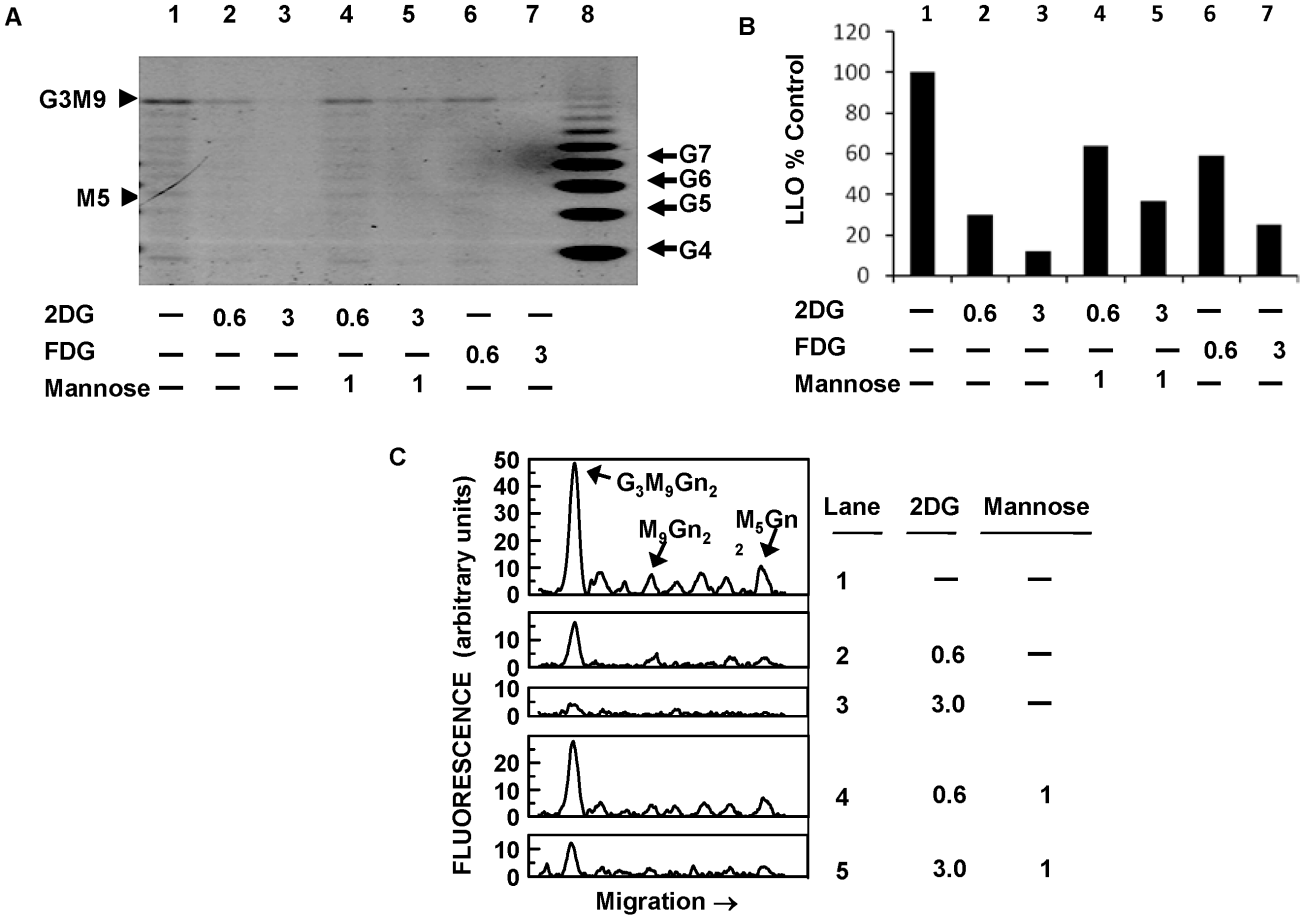
2-DG interferes with N-linked glycosylation by inhibiting lipid-linked oligosaccharide (LLO) assembly. **A**. Cells were treated with 2-DG with or without 1 mM mannose, and 2-FDG for 24 h, followed by extraction and fluorophore assisted carbohydrate electrophoresis (FACE) of LLOs. The standard oligosaccharides used in these studies are as follows: G4 to G7, glucose oligomers; G3M9, mature oligosaccharide (G_3_M_9_Gn_2_); M5, oligosaccharide intermediate (M_5_Gn_2_). 2-DG inhibited assembly of mature LLOs. Lane 1 =  untreated control. Lane 2 = 0.6 mM 2-DG; lane 3 = 3 mM 2-DG; Mannose reverted 2-DG inhibitory effects on LLO synthesis (lane 4, 2-DG at 0.6 mM + mannose, 1 mM; lane 5, 2-DG 3 mM + mannose, 1 mM). 2-FDG (lane 6: 0.6 mM; lane 7: 3 mM) treatment also decreased LLO synthesis, albeit at a lesser degree than 2-DG. Lane 8: glucose oligomer standards. **B**. The levels of mature LLOs were quantitated by measuring the fluorescence of G_3_M_9_Gn_2_-ANDS bands in each lane (arbitrary units), which is calculated by the percentage of band intensity in treated as compared with control samples. Bars represent the averages of single determinations in two separate experiments. **C.** To show minor LLO intermediates as well as the effects of mannose rescue more clearly (from figure 4A), electronically-generated traces of M_5_Gn_2_ through G_3_M_9_Gn_2_ in lanes 1–5 (from panel A) are displayed.

To directly demonstrate that 2-DG interferes with endothelial cell N-linked glycosylation, the effects of 2-DG on endothelial lipid linked oligosaccharide (LLO) synthesis were determined by fluorophore assisted carbohydrate electrophoresis (FACE). This non-radioactive method avoids the use of radioactive sugar precursors such as [^3^H]-mannose, the metabolism of which could be impaired by 2-DG treatment. As seen in figure 4A, B and C, 2-DG significantly reduced the formation of mature LLO (G_3_M_9_Gn_2_), at concentrations of 0.6 and 3 mM. Quantitative analysis (Fig. 4B) showed that 0.6 mM and 3 mM of 2-DG inhibited LLO formation by 70% and >80%, respectively. 2-FDG (a weaker inhibitor of N-linked glycosylation than 2-DG [18]), at a concentration of 0.6 mM, inhibited LLO synthesis by 40%, while at 3 mM, it inhibited LLO synthesis by about 75% (Fig. 4B). 2-DG interfered with the synthesis of both mature (G3M9Gn2) and minor LLO intermediates (e.g. M9Gn2 and M5Gn2), as shown by electronically generated tracings of the above LLOs in figure 4C. Co-treatment with mannose rescued 2-DG's inhibition of LLO synthesis at lower (0.6 mM), and higher (3 mM) concentrations of 2-DG, however the degree of rescue was less prominent at the higher 2-DG concentration. Consistent with the loss of LLO, total pools of neutral N-linked glycans were reduced by about 50% and 75% after treatments with 0.6 mM and 3 mM 2-DG, respectively (data not shown). Together with the results of figure 3, these data are consistent with the hypothesis that in endothelial cells, 2-DG acts primarily as an inhibitor of the N-glycosylation pathway rather than as an inhibitor of glycolysis.

**Figure 5:**
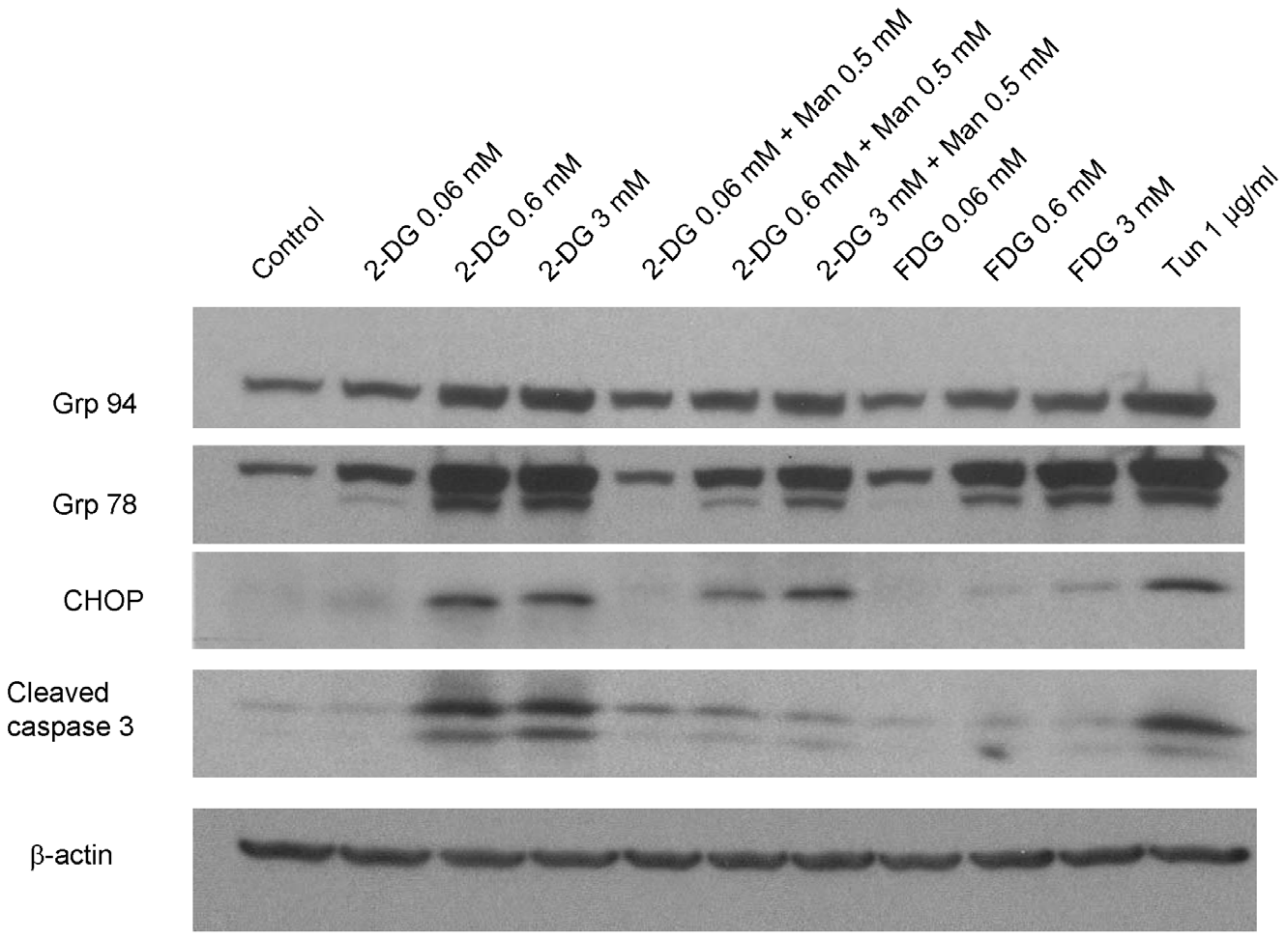
2-DG induces HUVEC unfolded protein response (UPR) and UPR mediated apoptosis. HUVECs were treated with 2-DG, with or without mannose, and 2-FDG for 24 hours, and immunoblotting was performed of cell lysates. 2-DG induced upregulation of Grp 94 (first panel) and Grp 78 (second panel) chaperone proteins and markers of the unfolded protein response. These effects were reversed by mannose (0.5 mM). CHOP/GADD 153, a transcription factor involved in ER stress mediated apoptosis, was potently upregulated by 2-DG (0.06, 0.6, and 3 mM) and partially reversed by mannose (third panel). CHOP/GADD 153 induction was associated with increased levels of cleaved caspase 3 in HUVECs treated by 2-DG (fourth panel). 2-FDG (0.06, 0.6, and 3 mM) induced a mild to moderate UPR response, especially at higher concentrations. However, CHOP/GADD 153 was not significantly induced, and levels of cleaved caspase 3 were not increased. Tunicamycin was used as positive control of the induction of UPR.

Interference with LLO synthesis impairs N-linked glycosylation, leading to accumulation of unfolded proteins within the endoplasmic reticulum (ER) and induction of an unfolded protein response (UPR) [38]. To investigate whether our 2-DG treatments triggered these events, HUVECs were exposed to 2-DG with and without mannose, and induction of UPR (as measured by its markers GRP 94 and GRP 78), as well as the activation of UPR mediated apoptotic pathways (as assessed by CHOP/GADD 153) were investigated by western blot. As shown in figure 5, 2-DG induced a dose dependent increase in GRP 94 and GRP 78, the latter being more prominent. Mannose partially reversed these effects. These effects were more prominent in 2-DG than 2-FDG treated HUVECs. UPR induction by 2-DG, (but not by 2-FDG), led to significant upregulation of CHOP/GADD 153 expression, and induction of endothelial cell apoptosis, as evidenced by increased caspase 3 cleavage (Fig. 5). Increased levels of CHOP/GAD D153 and cleaved caspase 3 were also found to be reversed by mannose.

**Figure 6:**
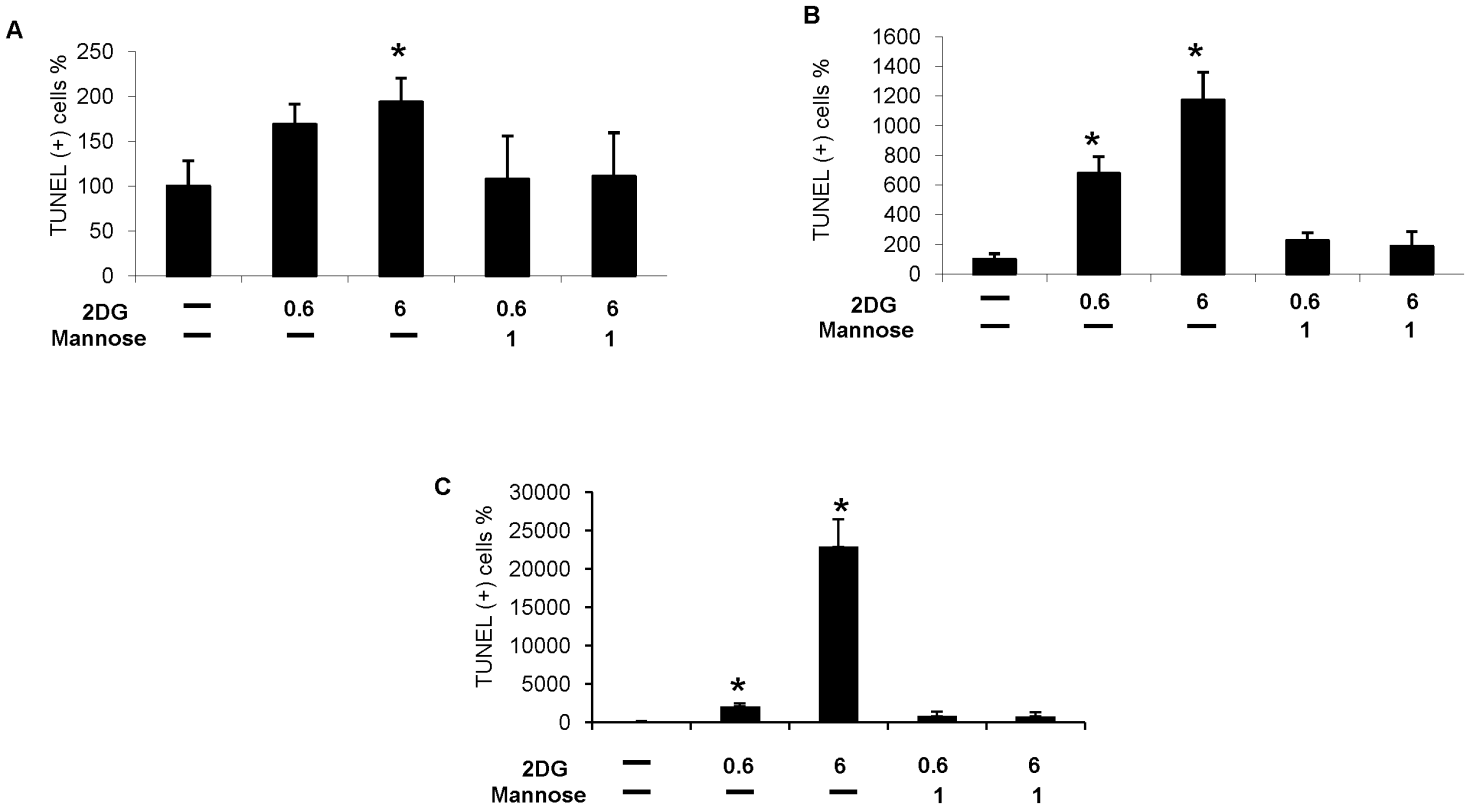
Induction of endothelial cell apoptosis by 2-DG and reversal by mannose. HUVEC cells in chamber slides were treated with 0.6 or 6 mM 2DG with or without 1 mM mannose and incubated for 24 (A), 48 (B), and 72 (C) hrs. Apoptosis (determined by TUNEL assay) was significantly induced upon 2DG treatment in a dose and time dependent manner at all time points tested. The pro-apoptotic effects of 2-DG were reversed by mannose treatment of HUVECs. Results are presented as percentage of TUNEL positive cells over total cells, normalized to untreated controls (+/− 95% CI). Experiments were performed in triplicate and repeated twice. * = p<0.05.

To further validate the findings that 2-DG induces endothelial cell apoptosis by the above mechanisms, apoptosis (by TUNEL assay) was assessed in HUVECs exposed to 2-DG with and without mannose. As shown in figure 6, treatment of HUVECs with 2-DG at concentrations of 0.6 and 6 mM was associated with significant induction of apoptosis, which could be detected at 24 hours (Fig. 6A) after exposure, and became prominent at 48 (Fig. 6B) and 72 (Fig. 6C) hours. As expected by the results described above, mannose (1 mM) reversed the pro-apoptotic effects of 2-DG at both concentrations and at all time points tested.

These results indicate that 2-DG induces HUVEC apoptosis predominantly by interfering with endothelial N-linked glycosylation, UPR induction, and activation of UPR-mediated apoptotic pathways.

### 2-DG inhibits angiogenesis *in vivo*

**Figure 7:**
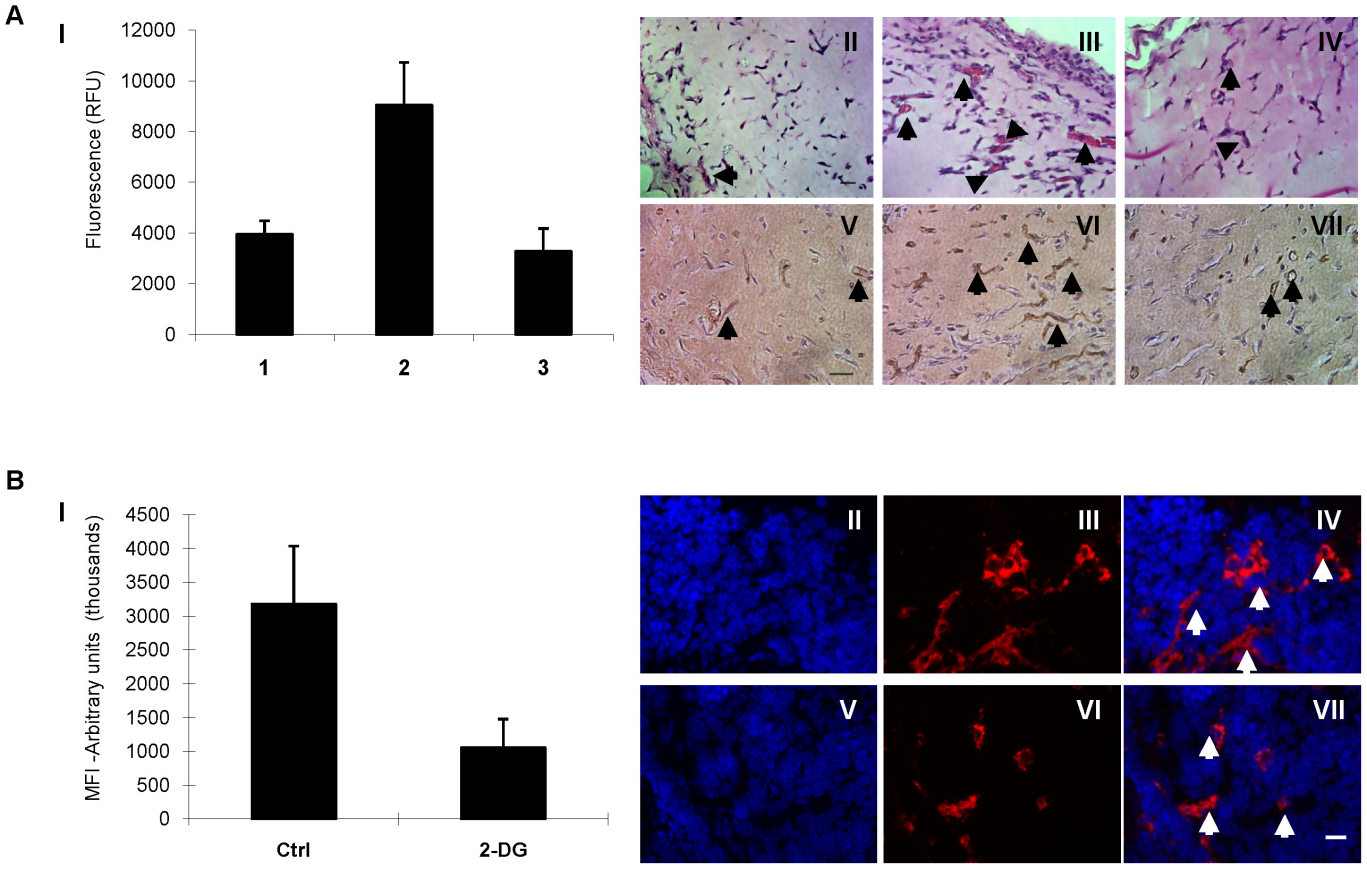
2-DG inhibits *in vivo* angiogenesis. **A**. Mice were injected with matrigel alone ((negative)control) or mixed with bFGF/VEGF and glucose (6 mM) or bFGF/VEGF and 6 mM 2-DG. Twelve days later mice were injected with FITC/dextran, plugs were removed and perfusion was determined by fluorescence. I. Matrigels mixed with bFGF/VEGF and glucose were associated with significant neovascularization (lane 2) compared to negative controls (lane-1). 2-DG, on the other hand, significantly inhibited *in vivo* angiogenesis, (lane 3 vs. 2). Bars represent means and 95% CIs of 5–6 mice per group. Matrigel plugs from additional mice were extracted for IHC analysis. Representative pictures of matrigel plugs stained with H&E (II, III, IV) and CD31 (V, VI, VII) are presented. II, V: Negative control. III, VI: positive control; IV, VII: 2-DG. Arrows indicate microvessels. Scale bar: 20 µm. **B.** The effects of 2-DG on tumor angiogenesis *in vivo* were evaluated in the *H_BETA_T_AG_* model of retinoblastoma. 2-DG was administered intraperitoneally (3 times per week, for 5 weeks) to tumor bearing mice as described in materials and methods. At the end of the treatment period, mice were euthanized and retinal tumors were extracted for analysis of tumor vasculature (lectin staining). I: Tumors in mice treated with 2-DG had a significant reduction of tumor microvessels (measured by quantification of lectin fluorescence) compared to saline treated mice (p<0.001). MFI =  mean fluorescence intensity. Bars represent means and 95% CIs of at least 4 independent samples per group. II–VII: Representative fluorescent pictures of retinal tumors from mice treated with saline (II, III, IV), and with 2-DG (V, VI, VII). II, V: DAPI staining. III, VI: Lectin staining III, VII: Overlay. Arrows indicate microvessels. Scale bar: 10 µm.

The *in vivo* relevance of 2-DG antiangiogenic effects was investigated in the in vivo murine matrigel plug assay [27], [33]. Mice were injected with matrigel alone without bFGF/VEGF (negative control), matrigel with bFGF/VEGF mixed with glucose (6 mM) “positive” control) or bFGF/VEGF and 2-DG (6 mM). Twelve days after matrigel implantation, mice were injected with FITC/dextran, plugs were removed and matrigel perfusion was determined by measuring matrigel fluorescence. *In vivo* angiogenesis in mice with plugs mixed with bFGF/2-DG had significantly less neovascularization, compared to the control group (Fig. 7A. I). *In vivo* angiogenesis was inhibited by approximately 64% in the 2-DG containing plugs (Fig. 7A. I, condition 3) compared to the control group (Fig. 7A. I, condition 2). Fluorescence counts (RFU) in the positive control group was 9047, while fluorescence in 2-DG exposed matrigels was 3281 (p<0.0001). These differences were observed histologically, by H&E staining (less red blood cell containing capillaries in the 2-DG group than the positive control, Fig. 7A. IV vs. 7A. III), as well as by CD 31 staining of microvessels, which were markedly reduced in the 2-DG treated groups (Fig. 7A. VII), compared to the control group (Fig. 7A. VI).

Boutrid et al. previously reported that systemic administration of 2-DG is associated with tumor delaying effects on the *LH_BETA_T_AG_* mouse model of retinoblastoma [34], with or without concomitant use of carboplatin [20]. In this model, transgenic mice typically develop ocular tumors with histological, ultrastructural and immunohistochemical features identical to those of human retinoblastoma [34]. Like the human counterparts, angiogenesis is a prominent feature in retinoblastoma tumors in this model and play an important role in tumor progression [20], [35], [39], [40]. Therefore, we examined the effects of systemically (IP) administrated 2-DG on *in vivo* tumor angiogenesis in this model. Mice were treated with intraperitoneal injections of 2-DG (500 mg/kg) as described in materials and methods. At 21 weeks of age, mice were sacrificed, retinal tumors were resected, and tumor vasculature was analyzed. As shown in figure 7B, a significant reduction in tumor microvessels (by lectin fluorescence staining) was observed in the group of mice treated with 2-DG (Fig. 7B. VI, VII) as compared with controls (Fig. 7B. III, IV). Mean fluorescence intensity (MFI) in the control group (arbitrary units -thousands-) was 3180.2 vs. 1058 (p<0.001). The above results strongly support the findings that 2-DG has potent *in vivo* antiangiogenic effects.

## Discussion

Targeting pathologic neovascularization is a clinically beneficial strategy for the treatment of cancer and angiogenesis-dependent diseases [2], [41], [42]. Antiangiogenic agents that are currently FDA approved or in clinical development, include monoclonal antibodies or multitargeted small molecule receptor tyrosine kinase inhibitors against VEGF and other endothelial pathways [2]. Other strategies being explored are direct vascular targeting, either by vascular disrupting drugs, or biological agents [2]. Here we provide evidence that interference of EC glucose metabolism with 2-DG may represent a new strategy for angiogenesis inhibition *in vitro* and *in vivo*.

Endothelial growth inhibition and cytotoxicity were induced by 2-DG at concentrations (0.6 mM) that did not significantly cause cytotoxicity in the non-endothelial tumor or non-tumor cells tested in this study (Fig. 1C). Previous reports have demonstrated that tumor cytotoxic concentrations of 2-DG *in vitro* are 4 mM and above [21], [29], [43]. One possibility to explain the unusual sensitivity of EC to 2-DG comes from previous reports in which it was shown that angiogenic growth factors (and hypoxia) significantly up regulate EC expression of glucose transporter-1 (GLUT-1) and glucose uptake [9], [11], [16], [37]. Moreover, a positive correlation between GLUT-1 expression and 2-DG sensitivity was reported by Maher et. al, in pancreatic cancer cell lines [43]. Our findings that 2-DG inhibits cell growth and induces cytotoxicity in HUVECs and HMVECs (Fig. 1C) at 72 hours, upon stimulation with bFGF alone (Fig. 1A, C), or with multiple angiogenic growth factors –such as VEGF, bFGF, IGF, etc, included in full endothelial growth medium (Fig. 1D; Figs. 2A, B, C; Figs. 3C, E), hold promise that the antiendothelial effects of this sugar analog may not be overcome by overexpression of alternative angiogenic pathways, a recognized mechanism of adaptive resistance to targeted (e.g. anti-VEGF) antiangiogenic agents [44].

*In vitro* antiangiogenic effects were also observed in endothelial cells exposed to 2-DG at earlier time points (24 hours or less), by the demonstration of inhibition of EC migration (Fig. 1D) and capillary formation (Fig. 2). Importantly, the finding that 2-DG inhibited predominantly *actively forming*, but not *already established* EC capillaries indicate that this sugar analog may preferentially disrupt endothelial cells during active angiogenesis. These findings suggest that 2-DG has the potential to act as a true antiangiogenic (preventing new vessel formation), rather than a vascular disrupting agent, and that its inhibitory effects occur at concentrations that may be clinically achievable [24]. The potent antiangiogenic effects observed *in vitro* also occur *in vivo*, as demonstrated in a murine angiogenesis model (matrigel plug assay, Fig. 7A) as well as in a transgenic model of retinoblastoma, where significant inhibition of tumor microvessels was observed, after systemic 2-DG administration (Fig. 7B). Lack of serious side effects of orally administered 2-DG has been reported by Raez et al. in a human phase I clinical trial of this agent in combination with taxotere, where no vascular serious adverse events related to 2-DG were observed [24]. Mohanti et al. also demonstrated safety and feasibility of the combination of oral 2-DG and radiation therapy in patients with supratentorial gliomas [25]. Recently, Stein et al. reported safety and feasibility results of 2-DG administration in subjects with advanced prostate cancer [23]. Pharmacokinetic data from the study by Stein et al. suggest that at the doses of 2-DG administered to patients, plasma concentrations of 2-DG may reach from 0.4 to 0.7 mM, which are very close to the “antiangiogenic” concentrations of this agent described in our study. These data support the concept that 2-DG may have the potential to target not only the tumor cell compartment, but also the tumor endothelium, and may significantly enhance the activity of other forms of anticancer therapy.

In microvascular endothelial cells, almost all the energy (ATP) derived from the catabolism of glucose is generated by glycolysis [4], [45]. At physiologic glucose concentrations, endothelial oxidative metabolism is inhibited, a phenomenon known as the Crabtree effect (inhibitory effect of glucose on mitochondrial respiration) [45]. Because glycolysis plays such an important role in endothelial ATP generation, we expected that the other glycolytic inhibitors tested would exert similar antiangiogenic effects as 2-DG, at equimolar concentrations. The finding that 2-DG had greater *in vitro* antiangiogenic effects than 2-FDG and oxamate, two potent glycolytic inhibitors, was therefore surprising. The demonstration that 2-DG's antiangiogenic effects were reversed by mannose (Fig. 3C, D, E), the sugar molecule involved in N-linked glycosylation, and the direct demonstration that 2-DG inhibits synthesis of endothelial lipid linked oligosaccharides (Fig. 4) indicate that the predominant mechanism of endothelial cytotoxicity and tube formation inhibition by 2-DG is interference of N-linked glycosylation. Previously we reported a similar effect of 2-DG in select tumor cell lines growing under normoxia and demonstrated that the endoplasmic reticulum stress induced by 2-DG led to an UPR activation of the apoptotic pathway (CHOP/GAD133). In that report it was demonstrated that mannose could reverse the cytotoxicity of 2-DG as well its interference on oligosaccharide synthesis and N-linked glycosylation, while mannose did not impair 2-DG uptake at the relevant concentrations. This previous data strengthens our conclusion that the predominant mechanism of 2-DG's endothelial cytotoxicity is via interference with N-linked glycosylation leading to UPR-mediated cell death.

The importance of surface glycoproteins and N-linked glycosylation on endothelial functions *in vitro* and *in vivo* has been reported [46], [47], [48]. Pili et al., showed that Castanospermine (CST), an alpha glucosidase inhibitor, which prevents the synthesis of complex oligosaccharides, inhibits bovine pulmonary artery ECs and bovine aortic ECs *in vitro*, and inhibits *in vivo* angiogenesis and tumor growth [48]. Martinez et al., demonstrated that N-glycosylation is critical for EC proliferation [46], [47], and that tunicamycin, an inhibitor of N-linked glycosylation, induces apoptosis in bovine adrenal microvascular endothelial cells [49]. The work presented here differs from the above studies in several aspects. First, the effects of low doses of 2-DG seem to preferentially affect actively forming capillaries, an effect that was not demonstrated with the above mentioned compounds. Second, CST and tunicamycin have been associated with potentially significant toxicity related to alteration in glycogen levels *in vivo* and neurotoxicity, respectively [50], [51], [52], [53]. This significantly limits their potential clinical development. On the other hand, 2-DG has displayed good bioavailability after oral administration, and safety has been demonstrated in early phase human clinical trials [23], [24], [25].

Notwithstanding our data which demonstrate that 2-DG effects on ECs are predominantly through interference with glycosylation leading to ER stress, it remains to be determined whether the anti-glycolytic effects of this sugar analog contribute to its overall antiangiogenic activity. Indeed, results presented in figure 3 suggest that glycolysis inhibition may play a role in 2-DG's growth inhibitory effect. This is based on our data which showed that 2-FDG (a more potent glycolytic and weaker glycosylation inhibitor than 2-DG [18]) and oxamate (pure glycolytic inhibitor) had non-significant cytotoxic effects (Fig. 3A), but did induce significant growth inhibition in ECs (Fig. 3B). The endothelial growth inhibitory effects of 2-FDG however, were significantly greater than that of oxamate, implying that interference with glycosylation (by 2-FDG but not oxamate) may be the more predominant mechanism for this effect. These findings further support the conclusion that inhibition of glycosylation is a necessary mechanism for 2-DG's cytotoxic and antiangiogenic effects. Studies to fully characterize the role of glycolysis and glycosylation on different steps of the angiogenic process *in vitro* and *in vivo* are in progress.

In conclusion, our findings underscore the importance of endothelial glucose metabolism, and provide a rationale to explore this target as a novel strategy for the treatment of angiogenesis dependent diseases.

## Supporting Information

Figure S1 2-DG inhibits endothelial cell growth in a dose and time dependent manner. 2-DG significantly inhibited bFGF induced HUVEC cell growth in a dose dependent manner at 24, 48 and 72 hours. Results (percent of control) are presented as the average of triplicate experiments and 95% confidence intervals. * =  p<0.05.
(0.45 MB TIF)
