# Supplementary figures and images for: Antiangiogenic Activity of 2-Deoxy-D-Glucose
Source: PLoS One. 2010 Oct 27;5(10):e13699. doi: 10.1371/journal.pone.0013699 (PMC2965179; doi:10.1371/journal.pone.0013699)

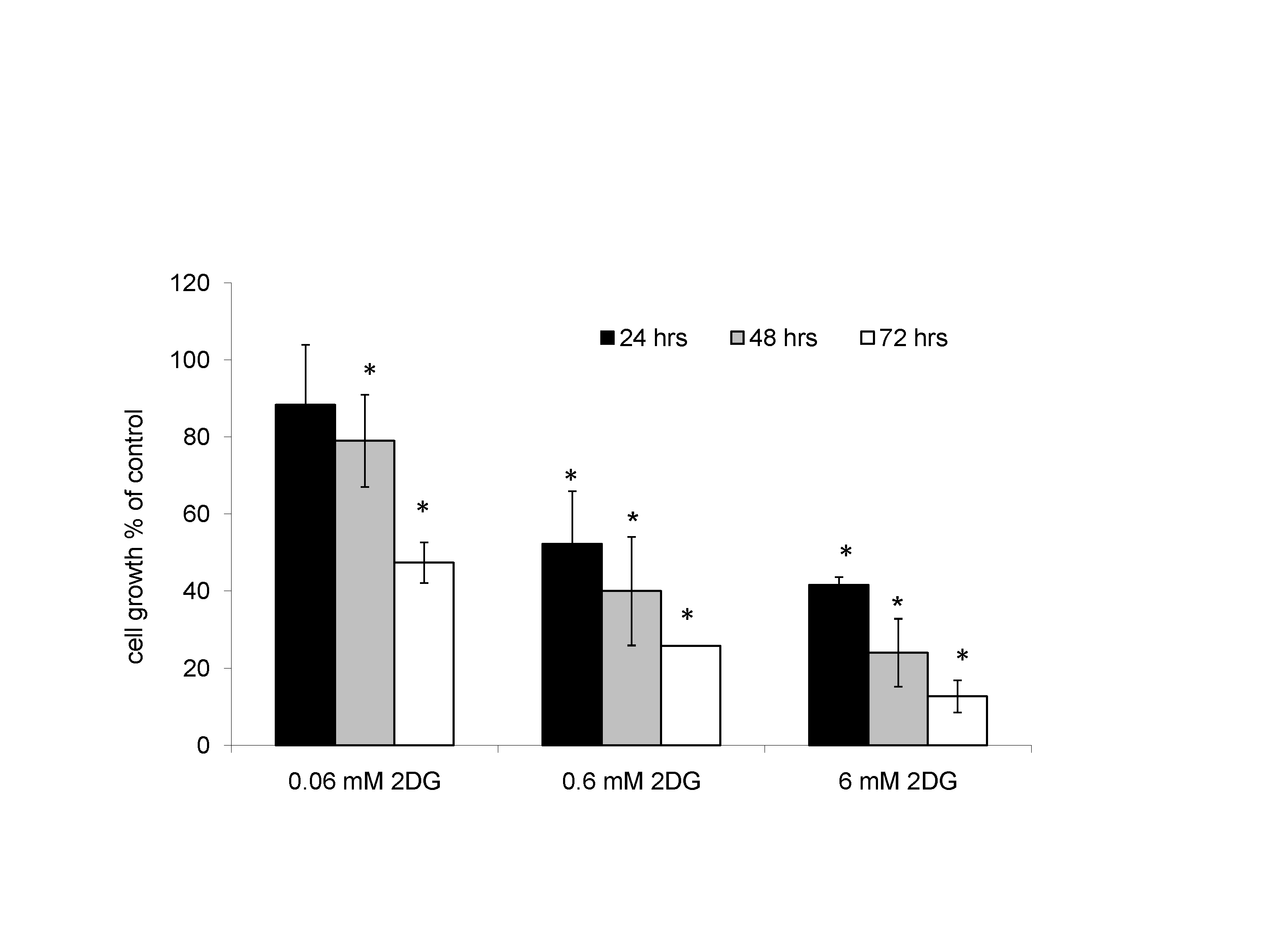

Supplement: Figure S1 — 2-DG inhibits endothelial cell growth in a dose and time dependent manner. 2-DG significantly inhibited bFGF induced HUVEC cell growth in a dose dependent manner at 24, 48 and 72 hours. Results (percent of control) are presented as the average of triplicate experiments and 95% confidence intervals. * = p<0.05. (0.45 MB TIF) [file pone.0013699.s001.tif]
